# Patient Perspectives on Gender Identity Data Collection in Electronic Health Records: An Analysis of Disclosure, Privacy, and Access to Care

**DOI:** 10.1089/trgh.2016.0007

**Published:** 2016-10-01

**Authors:** Hale M. Thompson

**Affiliations:** Section on Population and Behavioral Health, Department of Psychiatry, Rush University Medical Center, Chicago, Illinois.

**Keywords:** access to care, extended case method, disclosure, intersectionality, gender identity, privacy

## Abstract

**Purpose:** In 2015, the Centers for Medicare and Medicaid Services ruled that health organizations comply with additional requirements for electronic health records (EHRs), known as “Meaningful Use,” and develop the capacity to collect gender identity data. Research has established effectiveness of a two-step gender identity question to collect these data. This study examines transgender patient perspectives on the use of a two-step question and experiences with privacy and sensitive disclosures in EHRs and healthcare settings.

**Methods:** Four focus groups (*N*=30) were conducted in Chicago, Illinois in 2014–2015. Participants were asked to compare two intake forms—one with a two-step question and one with a single question—and discuss experiences with gender identity disclosure, privacy, and access to care. Narratives were transcribed verbatim to identify patterns and themes; the extended case method was used and grounded the data analysis process in the concept of intersectionality.

**Results:** Participants expressed appreciation for improved reliability and competencies that the two-part question may afford. Narratives reveal concerns related to patient privacy, safety, and access because of the contexts in which these data are collected and transmitted. Virtually all participants described situations whereby sensitive gender identity information had been involuntarily disclosed, misinterpreted, or abused, and safety and care were compromised.

**Conclusion:** Participants recognized the potential of the two-part question as a measurement and competency tool, but anticipated new privacy violations and involuntary disclosures. Narratives indicate that effects of sensitive disclosures may vary intersectionally, whereby white participants experienced lesser harms than their immigrant, HIV-positive, and black trans feminine counterparts. Discrimination and privacy violations may occur regardless of a two-part or one-part gender identity question, but increasing these sensitive disclosures within expanding EHR infrastructures may require a range of mechanisms that have flexibility across contexts to safeguard sensitive information and access to care.

## Background

**FIG. 1.**
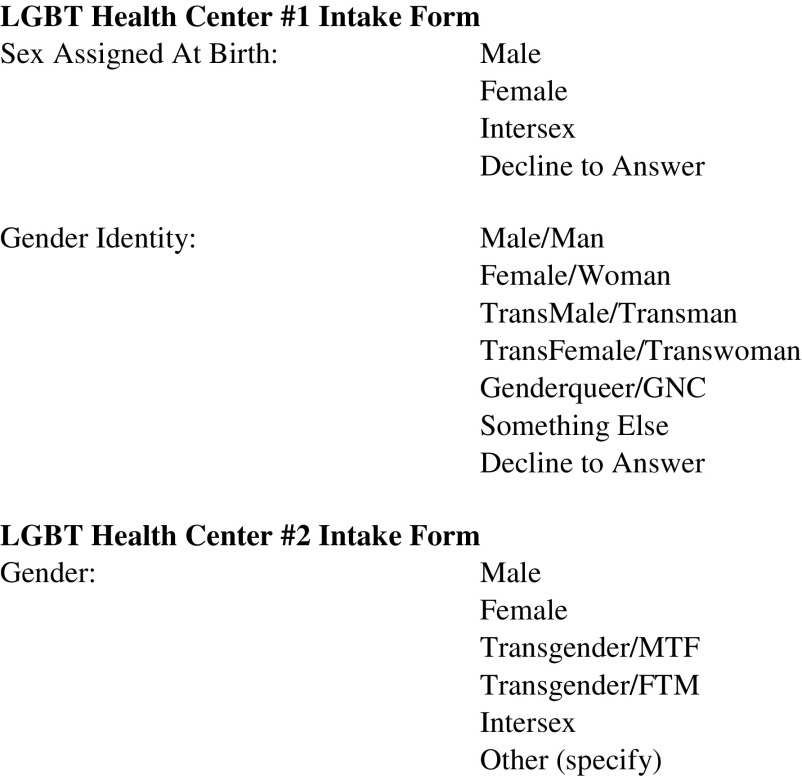
Assessment of gender identity on two LGBT clinic intake forms.

In October 2015, the Centers for Medicare & Medicaid Services and the Office of the National Coordinator for Health IT ruled that electronic health record (EHR) systems certified under Stage 3 of Meaningful Use must have the capacity to record, change, and access structured data on sexual orientation (SO) and gender identity (GI).^1^ Transgender* healthcare research^2^ has established the improved reliability of a two-step GI question and called for its standardization within EHR infrastructures.^3^ Such an algorithm (Fig. 1, LGBT Health Center 1) differentiates sex assigned at birth from gender and so more reliably identifies, enumerates, and tracks transgender patients as a population than single questions (Fig. 1, LGBT Health Center 2) that typically collapse sex and gender. How providers implement the two-step question within the EHR and the broader contexts of patient processes and provider workflows will impact its effectiveness as a data collection instrument and as an indicator of cultural competency. This article explores patient perspectives on the two-step question and experiences with patient privacy and disclosure in health records, healthcare settings, and related contexts; intersectional influences on access to care and GI disclosures are highlighted along with potential intervention points to improve privacy and facilitate access.

### The GI algorithm in healthcare settings

To better identify and enumerate transgender individuals, the recent ruling to encode GI categories into Meaningful Use-certified EHRs is consistent with calls in other domains of data collection. The Williams Institute has published a series of reports calling for use of a two-step question to ascertain GI in national health surveys on youth and adults and other surveillance systems.^4^ The contexts for both disclosure and data collection, storage, and transmission on surveys and surveillance systems differ from those of EHRs. While survey data are aggregated, sensitive disclosures within an individual's health record are often exposed to numerous parties besides the patient and clinician.^5,6^ For example, employers, pharmacists, and law enforcement have access to various aspects of health records as well as hospital registration staff, any of whom a patient may have to interact with repeatedly and may depend upon for essential resources.

LGBT advocates and researchers have suggested that standardizing and capturing GI in health records may more precisely enumerate a trans population, enable more and better research across and between disciplines, and expand our understandings of trans health at a population level.^3,7–10^ Another study highlights the feasibility of implementation of SO/GI data collection based on a short survey of primarily cisgender patients in four large community health centers (CHCs) that serve primarily LGBT and HIV-positive clients.^11^ Others contend that the two-step question will increase and normalize transgender visibility in the clinical setting, improving cultural competency and health outcomes.^12,13^

On the other hand, a case study highlights the challenges that arise when a clinic's EHR system interfaces with other clinical business associates, and the gender options and algorithms conflict. When the clinic transmitted data from the two-part question to external organizations, involuntary disclosure or misclassification occurred in two prominent ways: (1) the outside entity contacted and addressed patients by inferring pronouns/salutations based on sex, not gender, and (2) the clinic reported patients as transgender, who do not identify as trans (e.g., a patient who identifies as a man and whose sex assigned at birth is female), and reported nonbinary persons as cisgender (i.e., according to their sex assigned at birth).^14^

Others^15,16^ have shown how bureaucratic and administrative regulation of GI can create a visibility with coercive and violent effects on trans people. State agencies have various measures or definitions for sex classifications depending on an agency's mission or scope of services. Currah explains, “Sex classification serves different purposes at different city agencies—and that to put in place a single policy on sex reclassification across all city agencies would undermine the particular political rationalities at work in those policies.”^15^ While a trans person may count as male (i.e., their sex assigned at birth) according to federal guidelines of a homeless shelter, they may count as female (i.e., their current GI) for municipal public transit passes. For trans people in need of housing, transportation, and numerous other essential services, such conflicting classifications create possibilities for privacy violations, discrimination, and entanglement among bureaucratic, corporate, and administrative systems, which may define sex and gender classifications differently and specific to the administrative context.^15,17,18^

### Patient privacy, data security, and disclosure

When EHR systems emerged in the 1990s, Congress enacted the Health Insurance Portability and Accountability Act (HIPAA) of 1996 to address new privacy concerns related to technological shifts.^19^ Although widely viewed as grossly inadequate,^19,20^ the HIPAA Privacy Rule, effective in 2003, and the Security Rule, effective in 2005, mandated protections for patients' personal health information (PHI) and placed parameters around uses and disclosures of it without patient knowledge.^21^ As a corrective, Congress passed Health Information Technology for Economic and Clinical Health (HITECH) Act in 2009, which mandated more extensive regulation of disclosures of PHI.^21^

Historically and presently, privacy remains the province of those with privilege and resources, not persons on the social margins.^22,23^ Information security that is flexible and adaptive requires costly mechanisms such as encryption, data segmentation (i.e., securing segments of EHR data such that only specified types of users may access it), and anonymization techniques.^24,25^ As such, digital privacy of health records is nearly nonexistent particularly in under-resourced clinics where uninsured and publicly insured persons often receive care.^6,25^ While transgender persons with employer-based coverage may have access to well-resourced private providers and health insurance, poor or unemployed trans persons may access their care at transgender clinics housed in under-resourced public clinics.^26^

The literature around disclosure related to health and identity, such as HIV^27^ or SO,^21^ falls short with respect to GI. Only one study has examined how EHRs may complicate patients' privacy concerns around SO, not GI.^21^ The authors compare older and younger gay men's disclosure of SO in EHRs and found that nondisclosure of SO occurred more frequently among older gay men. The authors conclude that the younger men have less privacy concerns because they did not experience the stigma of HIV in the 20th Century and debuted sexually in the digital era, and express greater faith in digital privacy.^21^ This comparison enables identification of age-based disclosure norms, but renders unique disclosure strategies along other axes of marginalization, such as race and class, invisible.

### An intersectional framework

Intersectionality complicates understandings of identity and disclosure. Kimberlé Crenshaw developed intersectionality as an analytical tool to interrogate social contexts and highlight how the focus on a singular axis of identity creates erasures for those who occupy multiple socially oppressive locations and identities, such as black women.^28,29^ She notes, “Discrimination, like traffic through an intersection, may flow in one direction and it may flow in another. If an accident happens in an intersection, it can be caused by cars traveling from any number of directions and, sometimes, from all of them.”^28, p.149^ Intersectionality posits that identity categories and related axes of oppression are dynamic, mutually constitutive, and context specific rather than uniform and static.^30,31^ In other words, intersectionality may help identify ways in which a two-part GI question is effective for some in some settings and also how the data generated from it, and often shared in settings beyond the clinic examination room, introduce new opportunities for perceived discrimination, privacy violations, and involuntary disclosures.

## Methods

This research uses narrative analysis and the extended case method. The extended case method takes a reflexive approach that recognizes the researcher's relationship to the subject of study and identifies patterns, processes, anomalies, and paradoxes in the coded data to build on existing theory.^32^ Toggling between the micro and macro levels, this method is used to situate the focus group narratives within clinical, administrative, and policy contexts and draws upon intersectionality to understand patterns of disclosures and perceived discrimination.

### Study sample and data collection

The sample consisted of 30 trans and gender nonconforming (GNC) persons who participated in one of four confidential focus groups. Focus groups were digitally recorded in their entirety and transcribed verbatim. Transcripts and textual materials such as clinic intake forms and waiting room materials were analyzed and interpreted. The principal investigator conducted all focus groups and has a subject position similar to the participants in terms of GI. The IRB at University of Illinois at Chicago approved the research.

#### Focus groups

Focus groups were conducted in Chicago, Illinois, between October 2014 and January 2015. Recruitment occurred where trans persons tend to congregate such as local HIV prevention and primary care provider networks, social spaces, and one social media space, Facebook. Inclusion criteria for focus groups consisted of the following: (1) 18 years of age or older, (2) trans or GNC status for 1 year or more, and (3) the ability to speak English. Focus groups lasted ∼2–3 h and included sharing a meal or snack together. Participants received $30 compensation for their contribution to the research.

#### Focus group protocols

A facilitator guide with a fixed set of questions and probes was developed to discuss the intake process, clinical encounters, and various disclosure and nondisclosure experiences. Although mainly used for data analysis and interpretation, the extended case method was operationalized in the guide in at least two ways: (1) to query if and how identity and disclosure of identity shifted in different contexts, and (2) to query participants on their level of awareness of their provider's privacy policy. Participants also reviewed and discussed two sample intake forms from LGBT community clinics in the United States (Fig. 1). Intake forms collect personal demographic information during registration and become part of the medical record. Both forms queried for SO, preferred pronoun, and gender beyond the cisgender binary. These forms represented possible versions of standardized data collection instruments of GI. One used the two-step question, and the other used a single question.

### Analysis

Field notes were taken following focus groups, and memos were taken during the coding and analysis processes. While memos were used to identify emerging themes, field notes captured any surprises in the discussions, particularly those beyond the focus of questions. For example, “crosstalk care,” was an unexpected theme derived from memos and field notes and went beyond the scope of focus group questions. Crosstalk care reflects a peer-based form of care that occurred in each focus group, whereby participants shared successful strategies for overcoming a range of barriers to care. Transcripts were managed and coded using ATLAS.ti (Version 1.0.16; Scientific Software Development GmbH).

The coding process began with the random selection of one focus group. This process generated >100 codes. Focus group codes were organized into five categories: disclosure/privacy, GI classifications, quality of care, access to care/systems navigation, and crosstalk care. The remaining transcripts were then coded and analyzed iteratively for common themes. For example, the disclosure narratives elicited in focus groups were used to compare strategies reported in Stablein et al.'s article.^21^ The relationship between access and disclosure was compared across focus group participants' marginalized positions, such as assigned male at birth and an HIV-positive status. The patterns of experiences according to social positioning, identities and the social or clinical contexts were informed analytically by intersectionality.

## Results

### Focus group analysis

Focus group participants (*N*=30) represent a cross-section of trans and GNC persons in Chicago (Table 1). A total of 36 persons were screened; 35 were eligible. One person was excluded because he did not self-identify as trans or gender nonconforming, but was beginning to explore these identities. Five persons were unable to participate due to scheduling conflicts. Participants were in various stages of medical and/or social transitions, and their ages ranged from 19 to 73 with a median age of 30.5 years. In response to the eligibility question, “With what race and/or ethnicity do you primarily identify?,” participants identified primarily as black (33%), white (30%), Latina (27%), or Mixed race (10%). Of the nine Latina participants, three primarily identified as Mexican, two identified as Latina, two as Puerto Rican, one as Cuban, and one as Hispanic; none identified as black or white or indigenous. Seventy-seven percent reported sex assigned at birth as male, and gender identities expressed during eligibility screening included woman (40%), trans woman (37%), agender or nonbinary gender (10%), man (10%), and trans man (3%). Participants also said that their expressed gender identities shifted depending upon the context. Two young woman-identified participants were very hesitant to acknowledge having been assigned “male” at birth during the eligibility screening, but ultimately did. Although most were open with Facebook friends about their trans status, only two participants (7%) recalled actually having changed their Facebook gender marker to “trans” or “genderqueer,” or one of the other 50 new gender options that Facebook began to offer in 2014.

**Table 1.** **Sample Characteristics of Four Focus Groups, Chicago, IL, 2014–2015**

	Group 1, n=11	Group 2, n=7	Group 3, n=9	Group 4, n=3	Total, N=30
Sex assigned					
Male	11	3	9	0	23
Female	0	4	0	3	7
Current gender ID					
Male/man	0	2	0	1	3
Female/woman	4	2	6	0	12
Trans male	0	0	0	1	1
Trans female	7	1	3	0	11
Nonbinary/GQ/Agender	0	2	0	1	3
Race or ethnic ID					
Black/African American	1	0	7	2	10
Latina/Hispanic	8	0	0	0	8
White/Caucasian	0	7	1	1	9
Mixed race	2	0	1	0	3
Age (mean, SD)	(34.4, 13.6)	(28.7, 6.1)	(32.6, 15.9)	(46.0, 17.3)	
Jobless in last year					
Yes	9	5	3	3	20
No	2	2	6	0	10
Highest level education					
<High school graduate	2	0	3	0	5
HS diploma	4	0	0	0	4
Some technical school	0	0	1	0	1
Some college	4	2	3	1	10
Associates degree	1	1	0	1	3
College graduate	0	4	0	1	5
Graduate degree	0	0	2	0	2
Health insurance					
Yes	7	7	9	3	26
Public	6	0	6	1	13
Private	1	7	3	2	13
None	4	0	0	0	4

In terms of education, 66% reported at least a college-level education, and the same proportion of participants reported unemployment within the last 12 months. Eighty-seven percent reported having either private or public health insurance. The 13% reporting no coverage were all participants who speak English as a second language. Twenty-eight participants lived in the city, and two reported living in the suburbs. Twenty-nine received primary care in the city at the LGBT clinic, publicly funded community or HIV clinics, or clinics based at research hospitals. Only one participant, from the suburbs, reported not having a primary care provider, saying she received her care “here and there,” including emergency department, as needed (Focus Group 3).

#### GI classification and the two-step question

Overall, participants thought the two-step strategy (see, e.g., Fig. 1, LGBT Health Center 1) was an excellent way for a provider to identify those who occupy the social category of transgender, whether they actually identify as transgender. For example, some patients might have a trans history or experience, but identify as a man or woman. The two-part question captures patients who, similar to many of the focus group participants, often identify as men or women, but were assigned female or male, respectively, at birth. It also may capture those who identify as nonbinary, Two Spirit, agender, or genderqueer. Two participants who reported working in healthcare settings that prioritize trans patients noted that the use of the two-step question helps clinics report greater numbers of trans patients and increases access to related funding.

Participants were critical of the single question (see, e.g., Fig. 1, LGBT Health Center 2) for several reasons. Initially, some identified the conflation of sex and gender into one category as erroneous; one participant noted, “intersex” is not a gender. Some also criticized limiting the options to two binaries (i.e., male/female and female-to-male [FTM]/male-to-female [MTF]) and an offensive “other” term. One person noted that the transgender options, MTF and FTM, were outdated terms compared to trans man and trans woman, and the group agreed (Focus Group 2).

Finally, participants in all focus groups appreciated the “decline to answer” option for both questions and generally agreed that providers ought to always include that as a response option. As one nonbinary participant noted, “I do really like that they also have ‘decline to answer.’ Let's have a conversation about [my sex assigned at birth and gender identity] in the office in private” (Focus Group 2). Other participants wondered about the need to give this information at intake and if it was more appropriate to discuss with one's clinician depending upon the relevance to one's appointment.

#### Privacy, disclosure, visibility, and safety concerns

Although participants found the two-part question more substantively relevant, each group expressed concerns about answering these personal questions on an intake form, the data from which may be shared legally with any number of parties. Multiple instances of nondisclosure were given in every group, as were examples of involuntary disclosure. One participant, a Latina immigrant, described a more visceral violation of her privacy:

I was in the waiting room, and two nurses decided to discuss me. However, they failed to realize that they had the button for the PA system on. The whole damn waiting area and clinic could hear what they were discussing… I was without hormones for almost a year and a half, which caused havoc with my body and caused my nutrients to lower again when I started over again with Dr. XXX. It was bad (Focus Group 1).

Although this involuntary disclosure of GI was not tied to the two-part question, participants wondered if the new information from the two-part question might introduce additional opportunities for involuntary and unsafe disclosures in waiting rooms.

Participants' identities change, they said, depending upon the context and may not be relevant to their visit. Participants, particularly those who do not use LGBT clinics, said that, for privacy and safety reasons, they would not answer the sex assigned at birth question for fear of possible discrimination or even violence from intake staff or other patients. One young black woman, who refers to herself as unidentifiable as trans, described how a clinic worker threatened to call the police on her for using the women's restroom when he learned her legal status was “male.” She added,

At the end of the day, this is a registration form. You're giving this to the person who's registering you. For me, that could be a safety issue because how do I know after you—after I have identified myself as male or whatever the case may be—this is a very small world. We may be in public, and girl, you may point me out and tell this person, “Oh, girl. That's a man” (Focus Group 3).

Another young black participant shared a similar story where she disclosed her sex assigned at birth to the intake person at the emergency department and “before you knew it all these random people are popping into my room while I'm sitting there, waiting for the doctor. The janitor came in to change the trash. Three students came in. Four doctors came in” (Focus Group 3).

Others were concerned they might lose their jobs if this information reached their employer's human resources department by way of their insurance coverage. Many participants are not out as trans in their daily lives, especially at work. One black man reported that he was fired after his employer's human resources department received a health insurance invoice for his Pap smear. Another white man, also not out as trans at work, explained that providing his sex assigned at birth would exacerbate the anxiety he already feels with his employment-based health insurance. Although he was not fired, he said he risked one job when he had to out himself to human resources during his first week to determine which insurance plan would cover his trans-related healthcare.

Two white, nonbinary participants each said that they would hesitate to disclose GI information because clinicians do not know what to do with that information and tend to act on a set of assumptions. In their experience, doctors and staff very rarely use gender-neutral or gender-expansive pronouns and tend to assume that nonbinary persons want to take or should take hormones. These stereotypes can feel privacy violating when they are aired in front of others, when they have to be corrected, or when one has to explain that the stereotypes do not apply. One explained, “At this point I've given up on most medical providers. I've been trying to ask for [nongendered pronouns]. I do intend to change my name legally, which will make things much easier. The pronouns are still a whole other hurdle to get over” (Focus Group 4). Although the misgendering is alienating, the other participant said that they would rather “pass” as the gender associated with their sex assigned at birth than have to intervene on clinicians' erroneous assumptions about GI or their desire for medical transition, which tends to derail the central reason for their visits.

Some members of Focus Group 1 also reported preferring not to disclose their trans status when clinicians assume they were assigned female at birth. Rather than correct erroneous assumptions and face unpredictable responses, two participants said they instead volunteered answers to questions around their last menstrual cycle. “I just hit it off beautiful with this nurse, and it was wonderful. See, I always say, ‘Like a bird, blend’” (Focus Group 1). In this way, nondisclosure not only afforded safety from confrontation but also a perceived level of rapport that did not seem possible if she disclosed her trans status to the nurse. On the other hand, this privacy preserving, nondisclosure strategy could create barriers to care and negative health outcomes.

Although many participants wanted the option to “blend” if necessary, two healthcare workers, in different focus groups, discussed how visibility in healthcare settings works as part of a larger movement strategy for greater social acceptance of transgender people. One of them, who also positioned herself as older (i.e., late 50s), explained:

I'm not a female. I don't identify as a female. I'm a trans person, and I enjoy being a trans person. We are a contribution to society. So we should be recognized, just like a man or a woman… The only way to do it is to get more voice about it. That's why [I am a member of] the trans coalition and all the organizations that I belong to. For medical reasons, yes, I will put “transgender” (Focus Group 1).

Another participant, who also positioned herself as older (i.e., in her 70s), white, and a “late transitioner,” reduced the intake forms to finances and research data. She said, “By the way, the background on these [intake] forms, you can write a big dollar sign in the middle of them… in terms of grants. They need a lot of information so they can get that money” (Focus Group 3). Although not explicitly advocating coming out as part of a movement strategy, she was pointing to the financial and institutional power that providers seem to derive from their patients' disclosure of GI and related demographic data in these settings.

Some participants wondered if a clearer presentation of GI-related information in their EHR might help mitigate all the explaining they currently have to do during clinical encounters about their identity and the related care they need. Participants who used the LGBT clinic, which captures GI in the EHR, reported high clinician turnover, poor communication systems, long wait times, and short appointments. One white man who is a patient at the LGBT clinic said he had gone without hormones for several months because he felt so much anxiety around having to explain his identity and health needs to every new doctor even though his record contains his GI information. The other white man recalled of the same clinic,

I filled out this form several times, and the nurse comes in and confirms everything. And the next doctor interprets what was written down [in the EHR] in a totally different way, and he is misinterpreting this… How that happens with just a gender marker—several times—there was a lot of misgendering (Focus Group 2).

Participants expressed fatigue with having to reorient themselves frequently to new clinicians or related staff and subsequently reeducate them, in part, because the information in the EHR is not legible to clinicians and staff vis-à-vis the patient. Other participants expressed horror about seeing their legal names on their chart folders and laboratory stickers and wondered if clinician confusion comes from the way EHR systems display legal names and birth-assigned genders rather than the ones they live as.

Participants had mixed reviews of patient portals. Although some providers have launched patient portals through which patients can directly input their registration information, none of the focus group participants had registered as new patients at their provider through portals. Participants appreciated portal efficiency and that it can reduce interaction with human healthcare workers, while others questioned whether it afforded added privacy. One participant said, “I like [patient portals]. They are really convenient. You don't have to run back up to the doctor and get no test results, and do all that. Cause sometimes, they won't give you test results over the phone” (Focus Group 3). Participants also used portals to email their doctors for prescription refills, to schedule appointments, and to monitor when they need to return for blood draws. One participant expressed skepticism around the privacy and security of the portal:

I sent a message to one doctor and then it was a nurse who was working for them that saw it first and replied. Then they were figuring out where it was supposed to go after as opposed to just the doctor seeing it. I don't know how standard that is or who exactly is seeing those messages (Focus Group 4).

Another said she did not like using the portal: “I'm old fashioned. I like to pick up the phone and make my appointment and talk to a human being” (Focus Group 1). Healthcare with more and stronger human connections seems less alienating for some than others.

#### Administrative systems and influences on access to care

Although Meaningful Use strives to establish data standards in healthcare, EHR infrastructures and the numerous administrative systems with which they interface remain unique across providers and affiliated entities. Participants described a range of strategies to preserve their gender self-determination and access to resources despite noninteroperable administrative systems, but the strategies sometimes resulted in a trade-off of either gender self-determination or access to resources.

Attempting to make one's gender ID more consistent in state and healthcare administrative systems was one strategy reported. While it may have reduced the number of conflicting gender markers and the opportunities for misgendering, participants reported how changing one's legal name and gender marker in various systems can also create new barriers to care rather than eliminating them. One young black woman explained:

[The mis-gendering] didn't stop until I got female on my ID, and I just literally clapped down my ID and said, “The state recognizes me as female, so obviously, you need to recognize me as well.” I wish they would have a healthcare plan for me—’cause [now], they don't cover Delestrogen. I literally spent $225.00 for a vial of Delestrogen the other week (Focus Group 3).

Although her new gender marker forced the intake person to respect her and her GI, her public insurance stopped covering her medications when the gender marker changed. Another black woman said she preferred not to change her name and gender marker legally, and she has a very traditional, masculine name. “It's a matter of personal preferences… I haven't had my name changed yet… because I'm comfortable being me. I've worked for over 10 years, and I've never had any problems” (Focus Group 1).

Several participants across focus groups—all with public insurance—reported having problems in that they now had to pay out-of-pocket for costly medications after they legally changed their names and genders. Participants explained that the public insurance offered in Chicago, known as County Care, stops one's trans health-related coverage when gender markers or names are changed until patients can successfully appeal the decision, ostensibly to prove that the change is not for the purposes of committing fraud. Other participants, who had already changed their gender marker, also found themselves having to pay out-of-pocket once they signed up for their required ACA healthcare plans, although some did speak of having clinicians who knew how to adjust their diagnostic coding to avoid having coverage stopped.

An immigrant participant with chronic illness shared her experience with state bureaucracy and barriers to care. Although she changed her gender legally to female on her state ID many years ago, she let her ID expire when she was battling a life-threatening condition. After recovering, she attempted to renew her state ID only to be told that the Secretary of State now deems her male unless she could produce all the required paperwork to change her gender to female for the second time. Unable to reproduce that paperwork, she is now registered as male under her Medicaid plan, and her hormones are no longer covered.

Although not without challenges, trans men did not describe parallel ones around gender markers and administrative systems. One black participant reported that although he had never legally changed his gender from female to male, the Secretary of State's office staff voluntarily changed his ID's gender marker from an F to an M because they thought there had been an obvious coding error. He explained, “She looked at my ID. She goes, ‘Oh my God! They made a mistake on here.’ I said, ‘You're right.’ She goes, That should be an ‘M.’ I said, ‘I told them that!’ And she made it an ‘M.’ I've been riding the ‘M’ wave all the way” (Focus Group 4). Although he had never changed his name or gender legally, that “M” enabled him to marry, and subsequently divorce, his wife; he was also incarcerated with the male population in the local jail until some familiar trans women, also housed with the male inmates, outed him to jail officials. At that point, officials moved him to the jail infirmary rather than to the female unit of the jail.

A white participant reported that when he initiated care for a cold at a MinuteClinic, which are walk-in clinics within CVS drugstores, intake staff informed him that his insurance carrier must have made a clerical error because, in her check for his coverage, she saw that he is coded as female. Legally male for years, he did not know how she retrieved his information so quickly or why he would be registered as female in the database she checked. He said that the incredible anxiety that he felt would keep him from ever going to this type of walk-in clinic.

In addition to painful experiences with administrative system entanglement, participants described how some clinicians and staff go to great lengths to help navigate bureaucratic barriers to access care. In two focus groups, participants observed that the LGBT clinic had numerous empathetic employees that are of color and/or trans who “help you find a lot of stuff that you need… They help you get insurance [to enable you] to get the hormones… stuff that you need” (Focus Group 1). Beyond the LGBT clinic, participants described other skilled clinicians and staff—often immigrant or persons of color themselves—who had helped them access coverage for care and hormones with particular diagnostic coding that depended on legal gender markers, type of coverage, veteran status, HIV status, and other contingent factors that play into access.

## Discussion

This research prioritizes trans patient experiences with GI disclosure in healthcare settings and their perspectives on use of the two-part GI question to populate EHR databases across healthcare systems. The narratives highlight challenges with GI disclosure and data collection in healthcare, and an intersectional lens offers insights into how this process may differentially impact access to care, privacy, and other essential aspects of daily living. The varied experiences with disclosure raise questions about the acceptability of the two-part question as an identification and measurement tool; how can it address health disparities if the contexts in which the information is drawn and transmitted create new vulnerabilities for those at marginal intersections of identity? Unlike a survey or a census that enumerates and aggregates GI data, an EHR contains sensitive, individualized health information that may be shared in unanticipated ways. Although the two-part question may intend to improve competencies and health outcomes, noninteroperability with interfacing administrative systems and a lack of protective mechanisms around the information collected may create new vulnerabilities and deter these disclosures and accessing care.

Under the Affordable Care Act (ACA), access to trans healthcare has changed rapidly. Consistent with national findings on primary care,^33^ participants indicated that the current landscape includes increasing clinician turnover within clinics as well as patient turnover between clinics. This dynamic makes sensitive disclosures riskier because: (1) relationships with providers and clinicians are less stable and have less trust established, and (2) different clinicians often interpret the same gender-related information in the EHR very differently. With expanded insurance coverage, patients are choosing or having to seek care with non-LGBT primary care providers and health information requirements and data flows are also increasing substantially. The asymmetrical collection and nontransparent flows of patient information have multiplied with EHR implementation and the ACA.^5,34^ These asymmetries, whereby providers accumulate and circulate PHI in ways that are not always clear or transparent to patients, along with past experiences of healthcare discrimination (see also, e.g., Grant et al.^26^), appear to be associated with trans and GNC patients' hesitation about disclosing sex assigned at birth or, in the case of nonbinary participants—current GI—as well as accessing care at certain provider sites.

Although it may work well for many patients in some contexts, the two-part GI question may exacerbate harm for other transgender people. Before introducing opportunities to expose additional sensitive information, such as specification of sex assigned at birth, providers may need to devote resources to the protection of trans patients' sensitive personal information. Given that sensitive information is not always protected, patients may withhold sensitive information or avoid care altogether to minimize harassment, disrespect, and denial of services. Participants of color, immigrants, and HIV-positive persons relayed experiences where perceived discrimination and involuntary disclosures threatened their physical safety or altogether blocked access to care or medications. These harms deter disclosure of sex assigned at birth to anyone, but their clinician, and only if that information is relevant to their visit.

While trans feminine and nonbinary persons' identities were often challenged, particularly for those at the intersections of assigned male at birth, of color, HIV positive, and/or immigrant, administrative and clinical staff tended to privilege trans men's masculinity over any “F” catalogued in their information systems. System errors were assumed and amended. Transgender women did not report any such privileges, and trans men reported fewer challenges. The trans man's state ID was changed from F to M without engaging any formal procedures, while the trans woman's state ID was changed back to M from F even though she had completed the formal procedures and requirements. The trans inmate was housed in the jail infirmary when his sex assigned at birth was disclosed, whereas the trans females were housed with the cis male population. Not only are state-based rules for “sex classifications notoriously contradictory” across different agencies^15^ but also how they are enforced appear to vary along particular intersections of marginalization and privilege.

Identification of trans patients through a two-part question and acknowledgment of their current GI is only one step toward ensuring access to quality, respectful care. Participants have found allied clinicians and staff who honor their gender identities and help them navigate the information asymmetries of the current landscape. These human factors vary greatly across providers, and Section 1557 of the ACA may not resolve more immediate access barriers if at all. Given the apparent intersectional^28^ patterns of healthcare inequity and the vastly changing healthcare landscape, providers should consider, at the structural level, hiring and training more clinicians and staff with the requisite empathetic and administrative skill sets.

To further enhance data collection and access to care, stakeholders may consider the development of patient process and workflow mechanisms that increase levels of security and privacy. For example, patient portals, encryption, user-defined roles, and data segmentation of sex assigned at birth and legal names may help protect staff and patients alike from frequent misgendering and limit the footprint of this sensitive information. More research is needed to ensure that adding an algorithmic layer to capture gender identities within EHR infrastructures increases overall reliability and validity for ascertaining trans patients. A range of research in LGBT as well as non-LGBT CHCs and hospitals is necessary to observe and test security and privacy mechanisms before mandating standards.

The extended case method and intersectionality underscore the importance of social contexts and conceptual frameworks. While access to healthcare may vary intersectionally for patients, quality of care may also vary intersectionally for providers. A crucial problem to resolve is that security and privacy mechanisms such as patient portals, data segmentation, and a well-trained staff require significant human and financial capital, and the more financially marginalized providers and/or the non-LGBT ones may experience more barriers to effective implementation.

This analysis has limitations. It is a critical, narrative analysis of a sample of 30 trans and gender nonconforming persons in Chicago. While the sample is diverse, it is small and limited to self-report. All participants live in a major metropolitan area in a state that participates in the ACA's expansion of Medicaid. Additional studies in other geographic regions such as the South, where demographics differ and more states have opted out of the expansion, may offer new insights on the two-step algorithm's potential benefits and, especially, harms if implemented in places where trans persons already have greater difficulties accessing public accommodations. Similarly, a different researcher will bring a different subject position and lens to questions of GI disclosure and data collection in EHRs.

Focus group participants appreciated the nuance of the two-part question and the data that it could generate, but some of the response options as well as many problematic contexts for the data's collection and circulation generated concern for the consequences. The participants' experiences anticipate the ways that this identity data may siphon off, rather than expand or improve, their access to numerous resources in unpredictable and harmful ways. To address health disparities based on data from the two-step question, healthcare providers and EHRs must have better mechanisms to protect the privacy of patients and the security of data. These mechanisms will help ensure healthcare justice for trans and GNC patients, improving data collection and access to quality care, helping prevent discrimination based on GI, and giving more weight to the stated patient protections of Section 1557 of the Affordable Care Act.

## Notes

^*^ The terms transgender, trans, as well as gender nonconforming and nonbinary are used to describe individuals whose gender identity differs from the gender norms associated with their sex assigned at birth. The terms cisgender and cis are used to describe any individual whose sex assigned at birth aligns with their gender identity and expression. The article uses these terms with the understanding that they may not necessarily resonate with those to whom they are applied, but reflect a social positioning.

## Abbreviations Used

CHCs: community health centers
EHRs: electronic health records
GI: gender identity
HIPAA: Health Insurance Portability and Accountability Act
HITECH: Health Information Technology for Economic and Clinical Health
PHI: personal health information
SO: sexual orientation
